# Cerebellar Tonsillar Descent Mimicking Chiari Malformation

**DOI:** 10.3390/jcm12082786

**Published:** 2023-04-09

**Authors:** Rachel J. Park, Sunil Unnikrishnan, Joel Berliner, John Magnussen, Shinuo Liu, Marcus A. Stoodley

**Affiliations:** 1 Macquarie Health, Sydney, NSW 2109, Australia; rachel.park@mqhealth.org.au; 2 Macquarie Medical School, Macquarie University, Sydney, NSW 2109, Australia; sunil.unnikrishnan@mq.edu.au (S.U.); joel.berliner@mq.edu.au (J.B.); johnm@scan.com.au (J.M.); 3 Statewide Neurosurgical Service, Sir Charles Gairdner and Perth Children’s Hospitals, Perth, WA 6009, Australia; shinuo.liu@hdr.mq.edu.au

**Keywords:** Chiari I malformation, spontaneous intracranial hypotension, idiopathic intracranial hypertension, arachnoiditis, dural band, cysts, syringomyelia

## Abstract

Chiari I malformation has been defined as cerebellar tonsillar descent greater than 5 mm below the foramen magnum. Suboccipital decompression remains the mainstay of treatment for symptomatic patients. Other conditions sometimes have imaging features that mimic Chiari I malformation. These patients are at risk of misdiagnosis and mismanagement, including surgery that may be unnecessary or may even worsen the underlying condition. The aim of this study was to analyse a series of Chiari I malformation mimics and identify differentiating imaging features. The mimics are categorised as post-traumatic cranio-cervical junction arachnoiditis, dural band, spontaneous intracranial hypotension, idiopathic intracranial hypertension, and cysts. Better understanding of these conditions will assist with diagnosis and optimal management, including avoiding unnecessary surgery.

## 1. Introduction

Chiari I malformation is considered a congenital malformation of the paraxial mesoderm [1]. The anatomical features were originally described by Chiari [2], with cerebellar tonsillar descent as its characteristic feature. A common definition of Chiari I malformation is that one or both cerebellar tonsils are 5 mm or more below the basion-opisthion line as measured on a midline sagittal T1-weighted magnetic resonance imaging (MRI) scan [3]. Symptoms include occipital headaches, neck pain, disequilibrium, and gait disturbance. Patients may also present with lower cranial nerve dysfunction [4]. Surgical management, commonly in the form of suboccipital decompression, is indicated for individuals with symptomatic Chiari I malformation with or without syringomyelia [3].

Certain clinical conditions may mimic the symptoms and imaging features of Chiari I malformation. These patients are at risk of misdiagnosis and mismanagement. A misdiagnosis of Chiari I malformation has been made in patients with idiopathic intracranial hypertension [5,6,7] and spontaneous intracranial hypotension [8,9]. Subsequent posterior fossa decompression yielded no benefit or worsened the symptoms [7]. Solid tumours are another potential cause of tonsillar descent, but they are readily identified on standard imaging.

Here, we present a case series of patients to illustrate the imaging features that may help differentiate Chiari I malformation from mimics. These have been categorised under pathologies relating to post-traumatic cranio-cervical junction arachnoiditis, dural band, spontaneous intracranial hypotension, idiopathic intracranial hypertension, and cysts.

## 2. Materials and Methods

Fifteen clinical cases were retrospectively included in this study. The patients were seen at the Macquarie University Hospital in Sydney between 2012 and 2022. All patients had MRI features suggestive of Chiari I malformation. A whole spine MRI was conducted in all the patients, and it was confirmed that there were no other underlying pathologies, such as a tethered cord. The morphometric anomalies of the occipital bone and the posterior cranial fossa were not examined. The patients were categorised into five underlying conditions: post-traumatic cranio-cervical junction arachnoiditis, dural band, spontaneous intracranial hypotension, idiopathic intracranial hypertension, and cysts (Table 1). All patients were followed up 4 to 6 weeks post-surgery or -procedure. The study was approved by the Macquarie University Human Research Ethics Committee. 

## 3. Results

### 3.1. Group One. Post-Traumatic Cranio-Cervical Junction Arachnoiditis and Syringomyelia

**Figure 1:**
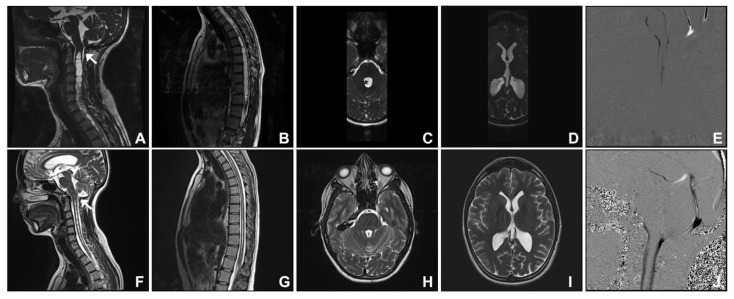
Chiari mimic: post-traumatic cranio-cervical junction arachnoiditis. A 26-year-old woman presented with a history of premature birth and a prolonged period in the neonatal intensive care unit. Investigations revealed what appeared to be a Chiari I malformation with a holocord syringomyelia (**A**,**B**). Additional magnetic resonance imaging (MRI) features include septa at the cervico-medullary junction consistent with cranio-cervical junction arachnoiditis ((**A**); white arrow), enlarged fourth ((**C**); * for enlarged fourth ventricle) and lateral ventricles (**D**), and a lack of dorsal cerebrospinal fluid (CSF) flow evident on phase contrast CSF flow studies (**E**). Posterior fossa decompression and shunting of the fourth ventricle to the spinal subarachnoid space was undertaken ((**F**); white arrow for shunt). At follow-up, there was full resolution of the symptoms in addition to reduction in syrinx size (**G**) and the fourth and lateral ventricles’ size (**H**,**I**), and established dorsal CSF flow on phase contrast CSF flow studies (**J**).

The two cases in this group were patients with traumatic events at birth (Case two) or in childhood with a significant head injury (Case one; see Video S1 for operative video). Both cases initially presented with imaging features of cerebellar tonsillar descent greater than 5 mm and syringomyelia. A 26-year-old female (Case two) presented with a 10-year history of left upper limb sensory disturbance and transient headaches. Investigations revealed an apparent Chiari I malformation with a holocord syringomyelia. There was a history of premature birth and a prolonged period in the neonatal intensive care unit. It was therefore suspected that cranio-cervical junction arachnoiditis (resulting from perinatal haemorrhage) and/or fourth ventricle outflow obstruction were the underlying pathologies rather than Chiari malformation. Additional imaging features included arachnoid septa at the cervico-medullary junction, an enlarged fourth ventricle, and limited cisterna magna with a lack of dorsal cerebrospinal fluid (CSF) flow evident on cardiac-gated cine MRI sequences and phase contrast CSF flow studies (Figure 1). At surgery, arachnoiditis at the cranio-cervical junction was evident in both cases, and a shunt was inserted from the fourth ventricle into the spinal subarachnoid space. In addition, the operation involved posterior fossa decompression, including removal of the superior part of the C1 posterior arch, and expansile duroplasty using a harvested pericranial patch graft. At follow-up, there was improvement in symptoms. A normal cisterna magna and CSF flow dorsal to the cerebellum were re-established. There was reduction in size of the fourth and the lateral ventricles and of the syrinx.

### 3.2. Group Two. Dural Band

**Figure 2:**
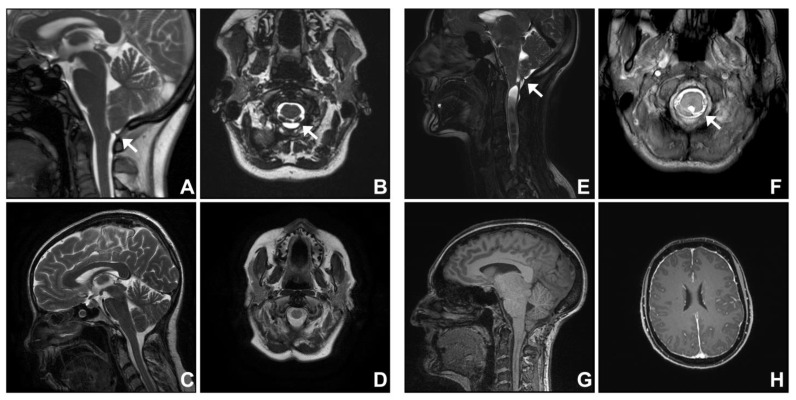
Chiari mimic: dural band. A 41-year-old woman (**A**–**D**) presented with a 4-year history of occipital headache and neck and bilateral upper limb pain. Cardiac gated steady-state free precession (SSFP or bFFE) imaging as well as thin-slice, heavily T2-weighted images showed cerebellar tonsillar descent, a thick dural band compressing the cerebellar tonsils ((**A**,**B**); white arrows), and a resultant reduction of cisterna magna (**A**,**B**). Decompression surgery and division of the dural band restored the cisterna magna to a normal size (**C**,**D**). A 49-year-old woman (**E–H**) presented with a 9-year history of hearing changes and sensory disturbance in the left upper limb and trunk. An MRI showed cerebellar tonsillar descent 7 mm below the foramen magnum up to the C1 arch with a communicating cervico-thoracic syrinx (**E**). In addition, compression of the tonsils at the cervico-medullary junction was evident from a distinct septation posteroinferior to the cerebellar tonsils ((**E,F**); white arrows) on cardiac-gated cine bFFE sequence (**E,F**). Following posterior fossa decompression, there was persistent cerebellar tonsillar descent, and imaging features were indicative of intracranial hypotension: prominent dural venous sinuses, diffuse pachymeningeal enhancement, pituitary gland enlargement, flattening of the pons, and dorsal beaking of the tectum (**G,H**).

Two patients with dural band at the cranio-cervical junction presented with symptoms and MRI features suggestive of Chiari I malformation. Both patients underwent posterior fossa decompression surgery including division of a dural band. In Case three (see Video S2 for operative video), the symptoms and imaging features improved post-operatively. The cisterna magna calibre was restored (Figure 2A–D).

A 49-year-old female (Case four) presented with a 9-year history of hearing changes and sensory disturbance in the left side of the trunk and the left upper limb. An MRI showed tonsillar descent 7 mm below the foramen magnum and a communicating cervico-thoracic syrinx. A distinct dural septum or band tethered the cerebellar tonsils at the cranio-cervical junction, distorting the tonsils and constricting the cisterna magna. This was best appreciated on the cardiac-gated cine MRI sequence (Figure 2E,F). At operation, a dural band at the tip of the tonsils was confirmed and resected. The operation involved posterior fossa decompression, including removal of the superior part of the C1 posterior arch, and expansile duroplasty using a harvested pericranial patch graft. Seven years following decompression, there were new symptoms, such as bilateral tinnitus, memory disturbance, and sensation changes in bilateral hands, despite a significant reduction of the syrinx (Figure 2E,G). In addition to persistent cerebellar tonsillar descent, there were imaging features indicative of intracranial hypotension. These included prominent dural venous sinuses, diffuse pachymeningeal enhancement, pituitary gland enlargement, flattening of the pons, and dorsal beaking of the tectum (Figure 2G,H). She was initially managed with a non-targeted blood patch. Subsequent MRI myelogram confirmed a CSF leak at T7-8, and surgical repair was conducted. Post-operative CT myelogram did not show a new site of CSF leak. However, pre-operative symptoms persisted, and she was lost to follow-up.

### 3.3. Group Three. Spontaneous Intracranial Hypotension

Six patients presented with chronic occipital headaches, with typical features of intracranial hypotension. These include postural headaches (worse when upright), neck pain, nausea, vomiting, cognitive deficit, and disturbances of vision, hearing, and balance. The average time to diagnosis was 4 years from the onset of the headaches. Tonsillar descent was demonstrated on MRI in all the patients. In addition, there were features of intracranial hypotension: pachymeningeal enhancement, brain sagging, venous sinus distension, and/or pituitary gland engorgement. To identify the site of the CSF leak, all the patients were examined with Computed Tomography (CT) myelogram, MRI myelogram, and/or digital subtraction myelogram. Three patients with no definitive imaging evidence of a CSF leak were treated with blood patches and obtained symptomatic relief. Three patients underwent surgical repair of a spinal CSF leak. All had substantial improvement in symptoms and follow-up imaging appearances.

**Figure 3:**
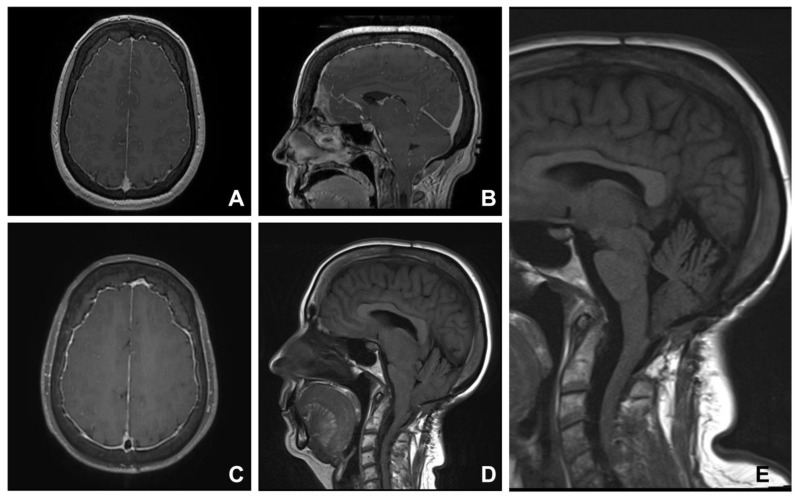
Chiari mimic: spontaneous intracranial hypotension. A 51-year-old woman was diagnosed with Chiari I malformation and syringomyelia and underwent posterior fossa decompression surgery at another institution. Worsening neurological symptoms led to further investigation for potential underlying spinal CSF leak. An MRI showed typical features of intracranial hypotension: diffuse pachymeningeal enhancement, pituitary gland enlargement, venous sinuses engorgement, and brain sagging appearance, an almost negligible pontomescencephalic angle, flattening of the pons, and cerebellar tonsillar descent (**C**,**D**). These features were present in the original pre-operative imaging (**A**,**B**). Surgical exploration at T8 confirmed a ventral spinal dural defect, and surgical repair led to an improvement in symptoms. In addition, post-operative imaging showed a restoration of pontomescencephalic angle, and interpeduncular and prepontine cisterns (**E**).

A 51-year-old female (Case nine) was diagnosed with Chiari I malformation and syringomyelia and underwent posterior fossa decompression surgery at another institution. Worsening neurological symptoms led to further investigation for a potential underlying spinal CSF leak. An MRI showed typical features of intracranial hypotension: diffuse pachymeningeal enhancement, pituitary gland enlargement, venous sinus engorgement, and brain sagging, an almost negligible pontomesencephalic angle, flattening of the pons, and cerebellar tonsillar descent (Figure 3). These features were present in the original pre-operative imaging (Figure 3A,B). MRI myelogram did not show extradural accumulation of a CSF leak. However, there was disc protrusion in the thoracic region around T8, raising the possibility of a CSF leak. Surgical exploration at T8 confirmed a ventral spinal dural defect, and surgical repair, with the use of fibrin sealant, led to an improvement in symptoms. In addition, post-operative imaging showed a restoration of the pontomesencephalic angle, and interpeduncular and prepontine cisterns (Figure 3E).

### 3.4. Group Four. Idiopathic Intracranial Hypertension

**Figure 4:**
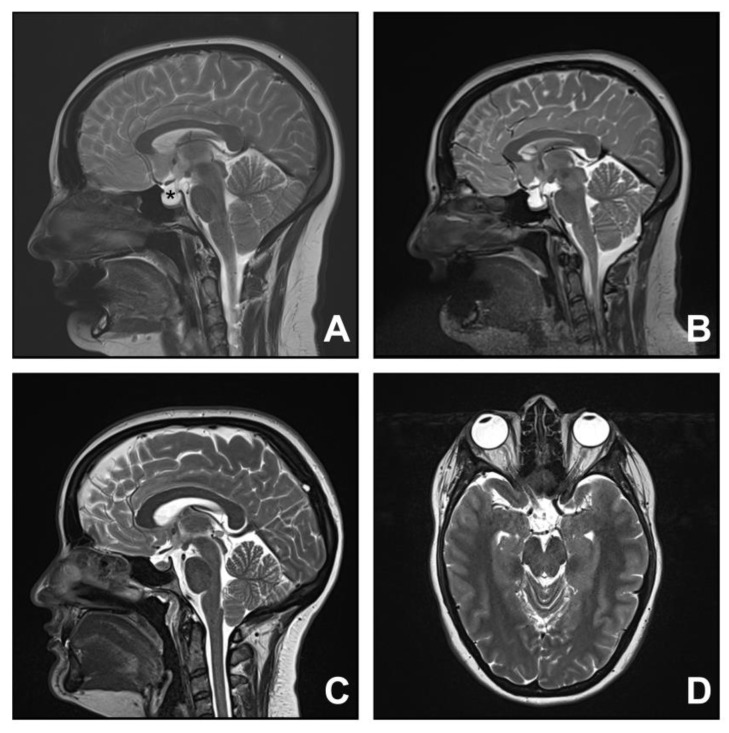
Chiari mimic: idiopathic intracranial hypertension. Two female patients with high body mass indexes (>30) (Case 11. (**A**,**B**); Case 12. (**C**,**D**)) presented with occipital headaches exacerbated by cough and MRI features suggestive of Chiari I malformation (**A**,**C**). In addition, there were concomitant imaging features of intracranial hypertension, such as empty sella ((**A**); * for empty sellar) and tortuosity and distension of the optic nerves (**D**). There was a persistent empty sella (**B**) post-posterior fossa decompression.

Two female patients with high body mass indexes (>30) (Cases 11 and 12) presented with occipital headaches exacerbated by cough and MRI features suggestive of Chiari I malformation. In addition, there were concomitant imaging features of intracranial hypertension, including empty sella, and tortuosity and increased CSF accumulation around the optic nerves (Figure 4). Lumbar puncture confirmed high opening pressures for both patients, 24 and 26 cm of water. Both patients were referred to a neurologist for medical management of the idiopathic intracranial hypertension. It was thought that one patient (Case 11) was symptomatic enough from posterior fossa crowding that posterior fossa decompression was warranted. Despite an uncomplicated operation and imaging improvement in the calibre of the subarachnoid space, the symptoms (headache and visual disturbance) worsened. She underwent CSF diversion by ventriculoperitoneal shunting (Miethke ProGAV 2.0 Adjustable Shunt System, B. Braun, Tuttlingen, Germany; valve set at 14 cm of water), which resulted in symptomatic relief.

### 3.5. Group Five. Cysts: Arachnoid, Choroid Plexus, and Cerebellum

Three patients in this group presented with symptoms and MRI features suggestive of Chiari I malformation secondary to arachnoid, choroid plexus, and cerebellar cysts. The patient with the arachnoid cyst underwent decompression and fenestration of the cyst, and the patient with the choroid plexus cyst underwent excision of the cyst. Both patients had full resolution of pre-operative symptoms, and MRI showed restoration of the cisterna magna. One patient with a cerebellar cyst had spontaneous resolution of the symptoms.

**Figure 5:**
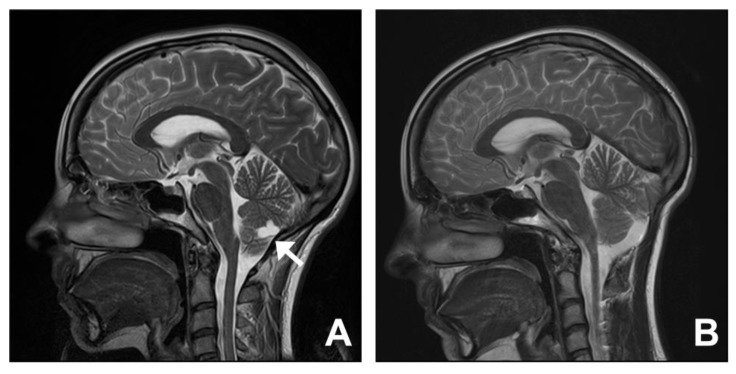
Chiari mimic: choroid plexus cyst. A 23-year-old woman presented with a 5-year history of headaches. An MRI demonstrated tonsillar descent and reduction in foramen magnum CSF space (**A**). There was an extra-axial cyst posteroinferior to the vermis between the cerebellar hemispheres ((**A**); white arrow). At surgery, the cystic lesion was found to be connected to the choroid plexus. The cyst was excised in a single piece and subsequent histopathology confirmed a choroid plexus cyst. At follow-up, there was full resolution of the symptoms. An MRI showed a restoration of the cisterna magna and no evidence of recurrence of the choroid plexus cyst (**B**).

A 23-year-old female (Case 14) presented with a 5-year history of headaches. Investigations revealed features suggestive of Chiari I malformation with tonsillar descent and a reduction in foramen magnum CSF space. An extra-axial cyst was also detected posteroinferior to the vermis. At surgery, the cystic lesion was found to be connected to the choroid plexus. This was excised en bloc and histopathology confirmed a choroid plexus cyst. In addition, the operation involved posterior fossa decompression, including removal of the superior part of the C1 posterior arch, and expansile duroplasty using a harvested pericranial patch graft. At follow-up, there was a full resolution of the symptoms. An MRI showed restoration of the cisterna magna and no evidence of cyst recurrence (Figure 5).

## 4. Discussion

Chiari malformation has been identified as the descent of one or both cerebellar tonsils 5 mm or more below the basion-opisthion line, as measured on a midline sagittal T1-weighted MRI scan [3]. However, it is clear that the pathology is much more complex, with different mechanisms of cerebellar tonsillar descent, including a small posterior fossa, spinal cord tethering, and cranial settling [10]. The standard definition of Chiari I malformation does not differentiate Chiari I malformation from other conditions that cause cerebellar tonsillar descent [3]. Other conditions with imaging features of tonsillar descent greater than 5 mm below the basion-opisthion line include post-traumatic cranio-cervical junction arachnoiditis, dural band, spontaneous intracranial hypotension, idiopathic intracranial hypertension, and cysts. Failure to recognise these conditions mimicking Chiari I malformation may lead to surgery that is ineffective for that condition or worsens the symptoms.

### 4.1. Cranio-Cervical Junction Arachnoiditis

Cranio-cervical junction arachnoiditis is caused by trauma, subarachnoid haemorrhage, meningitis, and previous posterior fossa operation [11,12]. Arachnoid pathology, such as arachnoid webs, thickening, and adhesions, is also commonly seen in individuals with Chiari I malformation [13,14,15]. The aetiology of arachnoid veils in Chiari I malformation is unknown. It has been proposed these entities result from repeated pulsation of the herniated tonsils against the cranio-cervical meninges [16]. The cases in this series with post-traumatic cranio-cervical junction arachnoiditis demonstrated a range of imaging features that suggest a diagnosis not consistent with Chiari I malformation. These include arachnoid septa at the cervico-medullary junction, an enlarged fourth ventricle, attenuated cisterna magna, lack of dorsal CSF flow, and tonsillar descent. Surgery may still be appropriate, but attention should be paid to the division of arachnoid adhesions, a fourth ventricle to subarachnoid shunt if there is obstruction of CSF outflow, as well as expansile duroplasty.

### 4.2. Dural Band

Dural band at the cranio-cervical junction has been described in individuals with Chiari I malformation [16,17,18]. A “tight dural band” or “thickened dura” was found flattening tonsils at the cranio-cervical junction intraoperatively in 40% of infants [18] and 10% of adults [17] diagnosed with symptomatic Chiari malformation. These studies considered the dural band to be an associated anatomical feature of Chiari I malformation. However, it has been suggested that trauma to the cranio-cervical junction may lead to the histopathological feature of a thickened dura, and the thickened dura alone could result in symptoms and imaging features of Chiari malformation [19]. Although standard posterior fossa decompression surgery with expansile duropasty and division of the dural band is effective in these cases, it is possible that division of the dural band alone may be sufficient. There is a paucity of evidence in the literature regarding this. In addition to the aetiology of dural band and its relationship with Chiari malformation, this is a subject worthy of further investigation.

### 4.3. Spontaneous Intracranial Hypotension

Spontaneous intracranial hypotension causes orthostatic headache attributed to low CSF pressure (<6 cm of water) [20]. Although it is clinically recognised by a typical orthostatic headache, the headache often becomes less posture-related with untreated chronic CSF leak [21]. There are many non-specific symptoms, such as neck pain, nausea, vomiting, cognitive deficit, decreased level of consciousness, or disturbances in vision, hearing or balance [22], which pose a challenge for appropriate investigation, diagnosis, and management. In addition, it may mimic Chiari I malformation on imaging [8,9]. This is consistent with international consensus, where there was high agreement (85.2%) that intracranial hypotension must be excluded for the diagnosis of Chiari I malformation [3]. Our Case nine shows the importance of recognising the imaging features of intracranial hypotension in an individual who was initially diagnosed and surgically managed for Chiari I malformation.

The specific imaging features of intracranial hypotension [22,23] include diffuse pachymeningeal enhancement, pituitary gland enlargement, venous sinus engorgement, subdural fluid collection, and/or “brain sagging” features, such as the descent of the third ventricular floor and mammillary bodies, a reduction in mammillo-pontine distance, flattening of pons against clivus, and cerebellar tonsillar descent [24,25,26,27]. Of these features, the sine qua non of intracranial hypotension is pachymeningeal enhancement on imaging [26]. However, it is postulated that such enhancement may decrease over time, especially in individuals with long-standing CSF leaks [28]. A recent study showed the slope of the third ventricular floor and pontomescencephalic angle as highly sensitive and specific features for the accurate diagnosis of intracranial hypotension in individuals with tonsillar descent, as opposed to the diagnosis of Chiari I malformation [25]. Recognition of imaging features of intracranial hypotension will avoid posterior fossa decompression surgery that may worsen the problem.

### 4.4. Idiopathic Intracranial Hypertension

Idiopathic intracranial hypertension is a syndrome consisting of clinical signs and symptoms of increased intracranial pressure, including headache, papilloedema, and elevated CSF pressure in the absence of ventriculomegaly and/or mass lesions [29]. Recent studies show the common occurrence of venous outflow obstruction in individuals with idiopathic intracranial hypertension [30,31].

Patients with idiopathic intracranial hypertension may present with cerebellar tonsillar descent mimicking Chiari I malformation [29,32,33], consistent with high agreement (96.3%) in a recent international consensus that it must be excluded from Chiari I malformation [2]. Such mimicking, together with the heterogeneous symptoms, leads to difficulty in differentiating between intracranial hypertension and Chiari I malformation. Correct diagnosis of intracranial hypertension in patients with concomitant cerebellar tonsillar descent is facilitated by the recognition of key imaging features. These include transverse sinus stenosis, empty sella syndrome (hypophysis–sella ratio of less than 0.5), and/or tortuosity of the optic nerve [34,35]. For instance, an MRI of Case 12 showed a tortuosity and increased CSF accumulation around the optic nerves in addition to the tonsillar descent (Figure 4D).

The co-existence of cerebellar tonsillar descent in individuals with intracranial hypertension raises a question of cause and effect. On the one hand, increased intracranial pressure in individuals with idiopathic intracranial hypertension may lead to tonsillar descent, mimicking Chiari I malformation. On the other hand, individuals with Chiari I malformation may have decreased CSF outflow from the fourth ventricle due to tonsillar descent, predisposing them to increased intracranial hypertension. Hence, the optimal initial surgical management is unclear. Is posterior fossa decompression or CSF diversion most appropriate?

Case 11 had persistent symptoms despite surgical decompression for symptomatic Chiari I malformation. She subsequently underwent insertion of a ventriculoperitoneal shunt to address intracranial hypertension confirmed on lumbar puncture. Studies have shown limited or no benefit from surgical decompression in individuals with intracranial hypertension and Chiari I malformation [5,6,7,36]. Therefore, presence of one of the imaging features of intracranial hypertension would warrant further investigation before consideration of surgical intervention for Chiari I malformation. Our approach for these patients is to decompress the Chiari I malformation first. The rationale for this approach is that underlying idiopathic intracranial hypertension can then be managed more readily, including safe lumbar puncture. We acknowledge that treating the idiopathic intracranial hypertension first is an alternative approach.

### 4.5. Cysts: Arachnoid, Choroid Plexus, Cerebellum

Space-occupying lesions, in particular arachnoid cysts in the posterior fossa, can mimic Chiari I malformation with cerebellar tonsillar descent [37,38]. It may be unclear whether the two entities, cysts and Chiari I malformation, are concomitant, or the cerebellar tonsillar descent is due to the mass effect of the cysts, like solid tumours. In asymptomatic patients with no neurological deficit, benign cysts, such as arachnoid and choroid plexus cysts, with associated imaging features of Chiari I malformation are managed conservatively [37]. Our Case 15, with a supracerebellar cyst with associated features of hydrocephalus and tonsillar descent, was managed conservatively.

Surgical management for symptomatic patients is warranted to improve the quality of life. Systematic evaluation of a case series by Wang et al. [38] demonstrated that with or without posterior fossa decompression, resection of space-occupying lesions yielded similar results in terms of the resolution of clinical symptoms and tonsillar descent. For Cases 13 and 14, in addition to fenestration and excision of arachnoid and choroid plexus cysts, respectively, posterior fossa decompression surgery was performed. Both patients had marked improvement in symptoms and post-operative imaging with restoration of the cisterna magna. There remains the question of whether posterior fossa decompression surgery is warranted for presumed acquired cerebellar tonsillar descent from mass effect of the cysts, or potential concomitant Chiari I malformation. Recognition of the posterior fossa cysts will guide surgical management.

### 4.6. Syringomyelia

Syringomyelia is a commonly associated feature of Chiari malformation [39]. In Chiari I malformation, there is no direct communication between a syrinx and the fourth ventricle [4,40].

Syringomyelia is also associated with other conditions that mimic Chiari I malformation, such as arachnoiditis and intracranial hypotension [40,41,42], as well as spinal cord injury, tethering, infection, and congenital malformation [4]. In this series, a syrinx was present in patients with long-standing underlying conditions, including congenital dural pathology, birth- and childhood-trauma-related arachnoiditis, and intracranial hypotension. There was a direct connection between the syrinx and the fourth ventricle via the central canal in all cases. The communicating syrinx was likely caused by obstruction of the fourth ventricle outlets [40,43] from arachnoiditis or dural band, or from cerebellar tonsillar descent. Outflow obstruction may lead to the direct flow of CSF into the syrinx via a patent central canal based on the proposed theory of the “water-hammer” effect [43], and this may be accentuated by forceful CSF flow down to the syrinx from increased central venous pressure during the Valsalva manoeuvre [44,45]. The communicating syrinx reduced in size in all our cases after surgery. This includes Case four, who had concurrent congenital dural pathology and intracranial hypotension, along with cerebellar tonsillar descent. She had a significant reduction of the syrinx upon restoration of the CSF outflow at the cranio-cervical junction despite persistent features of intracranial hypotension, including tonsillar descent. Imaging evidence of a direct communication between a syrinx and the fourth ventricle is highly suggestive of a Chiari I malformation mimic.

## 5. Conclusions

Several conditions that mimic Chiari I malformation with features of cerebellar tonsillar descent have unique and distinctive imaging features. Recognition of these features will assist with prompt and correct diagnosis of Chiari I malformation. Furthermore, this will enable the delivery of optimal management, which includes avoiding unnecessary surgeries that may worsen symptoms and the underlying condition.

## Figures and Tables

**Table 1:** Summary of five conditions that mimic Chiari I malformation.

Case	Age (Years)	Sex	Presentation	Duration (Years)	MRI (Additional Features to Tonsillar Descent)	Management	Outcome
**Post-traumatic cranio-cervical junction arachnoiditis**							
1	62	F	Balance and gait deterioration	10	Septa at cervico-medullary junction, syringomyelia	Posterior fossa decompression and insertion of fourth ventricle to spinal subarachnoid shunt	Stable
2	26	F	Cough-induced left upper limb sensory disturbance	10	Septa at cervico-medullary junction, holocord syringomyelia	Posterior fossa decompression and insertion of fourth ventricle to spinal subarachnoid shunt	Improved
**Dural band**							
3	41	F	Occipital headache, neck and bilateral upper limb pain	4	Septation at cranio-cervical junction, inferior to cerebellar tonsil	Posterior fossa decompression	Improved
4	49	F	Left trunk and upper limb sensory disturbance	9	Septation at cranio-cervical junction (inferior to cerebellar tonsil), cervico-thoracic syringomyelia	Posterior fossa decompression	Stable; Lost to follow-up
**Spontaneous intracranial hypotension**							
5	49	F	Headache	8	Pachymeningeal enhancement Brain sagging	Blood patch	Improved
6	73	F	Headache	1	Brain sagging	Blood patch	Improved
7	47	F	Headache	5	Brain sagging	Thoracic laminectomy for CSF leak	Improved
8	52	F	Headache	2	Pachymeningeal enhancement, pituitary gland engorgement	Blood patch Thoracic laminectomy for CSF leak	Improved
9	51	F	Headache	8	Pachymeningeal enhancement, venous sinus engorgement, brain sagging, syrinx	Multiple surgical decompressions Shunt insertion and revision for syrinx Thoracic laminectomy for CSF repair	Improved
10	68	F	Headache	2	Pachymeningeal enhancement, venous sinus engorgement, brain sagging, syrinx	Blood patch	Stable
**Idiopathic intracranial hypertension**							
11	26	F	Occipital headache Visual disturbance	5	Empty sella, CSF around optic nerves	Surgical decompression Ventriculoperitoneal shunt insertion	Improved
12	30	F	Occipital headache	8	Tortuosity of optic nerve	Medical management	Not applicable
**Cysts—arachnoid, choroid plexus, cerebellum**							
13	57	M	Headache Visual changes	1	Arachnoid cyst	Surgical decompression and fenestration of cyst	Improved
14	23	F	Headache	5	Extra-axial cyst	Surgical decompression and excision of cyst	Improved
15	61	F	Headache	1	Supra-cerebellar cyst	Expectant management	Improved

## Data Availability

No new data were created or analyzed in this study. Data sharing is not applicable to this article.

## Supplementary Materials

The following supporting information can be downloaded at: https://www.mdpi.com/article/10.3390/jcm12082786/s1, Video S1: Post-traumatic cranio-cervical junction arachnoiditis; Video S2: Dural band.
